# Gastropexy-Assisted vs. Gastropexy-Free Introducer Percutaneous Endoscopic Gastrostomy: A Prospective Randomized Trial

**DOI:** 10.1055/a-2942-4014

**Published:** 2026-09-07

**Authors:** Claudio C. Conrad, Pernilla V. Conrad, Julia-Sophie Herrmann, Arne Lassen, Konrad Aden, Stefan Schreiber, Mark Ellrichmann

**Affiliations:** 1 Interdisciplinary Endoscopy, Department of Internal Medicine 1 15056 University Hospital Schleswig-Holstein, Campus KielKielGermany; 2 Department of General, Visceral, Thoracic, Transplantation and Pediatric Surgery 54186 University Hospital Schleswig-Holstein, Campus KielKielSHGermany; 3 Department of Internal Medicine, Gastroenterology, Hepatology, Endocrinology and NephrologyUniversity Hospital OldenburgOldenburgNDSGermany

**Keywords:** endoscopy upper GI tract, gastrostomy and PEJ, malignant strictures, benign strictures

## Abstract

**Introduction** Percutaneous endoscopic gastrostomy (PEG) is a well-established and low-complication procedure for providing long-term enteral nutrition. In cases of oropharyngeal or esophageal stenosis, PEG can be safely performed using an introducer PEG. Until recently, the only established introducer PEG involved the use of gastropexy sutures. In June 2021, an alternative technique received regulatory approval. This method employs introducer PEG without prior gastropexy, securing the stomach to the abdominal wall using a tire-shaped balloon (FLOCARE DirectPEG). To date, no studies have directly compared these two techniques.

The aim of this study was to prospectively evaluate and compare the procedural efficiency and clinical outcomes of gastropexy-assisted introducer PEG (gPEG) versus gastropexy-free introducer PEG (dPEG).

**Patients and Methods** This prospective, randomized trial included patients with a confirmed indication for enteral nutrition via PEG. Eligible patients were randomized in a 1:1 ratio to receive either gPEG or dPEG. The primary endpoint was PEG placement time, measured from local skin incision to successful intragastric positioning of the tube. Secondary endpoints included total procedure time, technical feasibility, adverse events, and functional tube patency over a 30-day follow-up period.

**Results** A total of 37 patients were enrolled, with 22 patients (59%) assigned to the gPEG group and 15 patients (41%) to the dPEG group. PEG placement time was comparable between the groups (260 ± 109 s vs. 265 ± 145 s; *p* > 0.05). However, the procedural performance of gPEG was rated significantly better than that of the dPEG system (mean score 1.5 vs. 3.0; *p* < 0.01). In two patients (13%) in the dPEG group, the procedure had to be discontinued due to technical issues with tube deployment. Minor postprocedural complications occurred in 2 patients (9%) in the gPEG vs. in 6 patients (40%) in the dPEG ( *p* < 0.01). No major complications were observed. During the 30-day follow-up, tube dislocation was documented in 2 patients (9%) in the gPEG group compared to 5 patients (33%) in the dPEG group ( *p* = 0.0953). Tube patency was maintained in 100% of cases. Due to emerging clinically relevant differences in procedural feasibility and complication rates, the study was terminated prematurely.

**Conclusion** In this study, the established gPEG demonstrated superior ease of use and a significantly more favorable safety profile. Based on these findings, the conventional gastropexy-assisted introducer PEG remains the preferred approach.

## Introduction

PEG is a well-established and widely used procedure for providing long-term enteral nutrition, particularly in patients with insufficient oral intake. Due to its minimally invasive nature, cost-effectiveness, and favorable safety profile, PEG has become a standard approach for nutritional support. However, despite its overall safety, PEG placement is associated with a relevant rate of adverse events (AEs), ranging from 4.9% to 23.8%, although the majority of these complications are minor and manageable. Consequently, PEG is among the endoscopic procedures with relatively high reported overall complication rates, largely reflecting the frequency of minor complications rather than severe adverse outcomes. 1 2 3

Two principal methods are employed for PEG tube placement: the pull-through (ptPEG) technique and the introducer PEG. The ptPEG involves transoral passage of the tube and is widely regarded as the standard approach. However, its applicability is limited in patients with high-grade stenosis or obstruction of the upper gastrointestinal tract, often due to malignancies. Furthermore, transoral manipulation during the pull-through procedure has been associated with the risk of tumor cell seeding into the PEG tract, 4 prompting concern particularly in oncologic settings with a curative therapeutic approach.

To overcome these limitations, the introducer PEG with gastropexy was developed (gPEG). Traditionally, this approach involves two gastropexy sutures placed via a designated fixation device, followed by direct trocar insertion through the abdominal wall. Recent data suggest significant advantages of this method over the pull-through approach, including a reported 90% reduction in major complications and a 50% reduction in minor complications mainly as infectious complications. 3 The direct puncture technique is particularly valuable in patients with obstructive tumors or altered upper GI anatomy, offering a safe and effective alternative where the ptPEG is contraindicated.

In an effort to combine the advantages of established techniques, hybrid-PEG approaches, which integrate the pull-through method with gastropexy, have been introduced. These methods aim to enhance tube stability and reduce complication rates typically associated with standard PEG placement, while maintaining the procedural familiarity and efficiency of the pull-through route. Early data suggest a favorable safety profile, particularly in high-risk patient populations; however, comprehensive comparative studies remain limited. 1

While gastropexy has been shown to increase procedural safety, its application is technically demanding and time intensive. This can be a significant drawback, especially in critically ill patients, where minimizing procedural time is essential to reduce stress, time of conscious sedation, and potential peri-interventional risk.

To address this limitation, a novel introducer PEG system (dPEG) (FLOCARE DirectPEG, Nutricia Milupa, Frankfurt, Germany) was developed and received CE certification in June 2021. This new system eliminates the need for gastropexy sutures by employing a balloon-based internal fixation mechanism. The design aims to simplify the procedure, shorten intervention times, and reduce trauma-related complications, such as local pain or infection. By circumventing both gastropexy and transoral tube passage, this device theoretically preserves the key advantages of traditional introducer PEG, while offering a potentially faster, less invasive alternative.

Despite its promising design, real-world data on the dPEG technique, particularly in terms of feasibility and short-term safety outcomes, are currently lacking.

Accordingly, this prospective, randomized study was initiated to systematically compare the new gastropexy-free introducer PEG (dPEG) system with the established gastropexy-assisted method (gPEG).

## Material and Methods

### Study Outline

This study was conducted as a prospective, randomized controlled trial aimed at evaluating the feasibility and AE rates of a novel dPEG (FLOCARE DirectPEG, Nutricia Milupa, Frankfurt, Germany) compared with an established gPEG system (Freka Pexact II, Fresenius, Bad Homburg, Germany). It was carried out in a real-world setting within routine clinical practice at a single tertiary care center. All procedures were conducted in accordance with the ethical standards outlined in the Declaration of Helsinki. Ethical approval was obtained from the Ethics Committee of the Medical Faculty of the Christian-Albrechts-University, Kiel, Germany (reference number: D590/21). Furthermore, the study was prospectively registered with the German Clinical Trials Register (Deutsches Register Klinischer Studien, DRKS; registration number: DRKS00027256). Written informed consent was obtained from all participating patients prior to enrollment. The study forms part of a doctoral thesis conducted by J.S.H. and received research support from Nutricia Milupa, Frankfurt, Germany.

### Patients and Randomization

Patients were eligible for inclusion if they had an independent clinical indication for PEG placement and met at least one of the following criteria: presence of an esophageal stricture, a diagnosis of head and neck cancer, or esophageal cancer. Additional inclusion criteria were age over 18 years and provision of written informed consent. Patients were excluded from the study if they had an American Society of Anesthesiologists (ASA) physical status classification greater than III, clinically significant ascites, uncorrected coagulopathy, or any uncontrolled infection at the time of enrollment.

After eligibility assessment and informed consent, patients were randomized in a 1:1 allocation to one of two study arms by using a closed-envelope method with predetermined, concealed allocation sequences prepared in a random order:

- Group A underwent PEG placement using the established gPEG system (gPEG group)
- Group B received PEG placement using the novel gastropexy-free dPEG system (dPEG group)

**Fig. 1:**
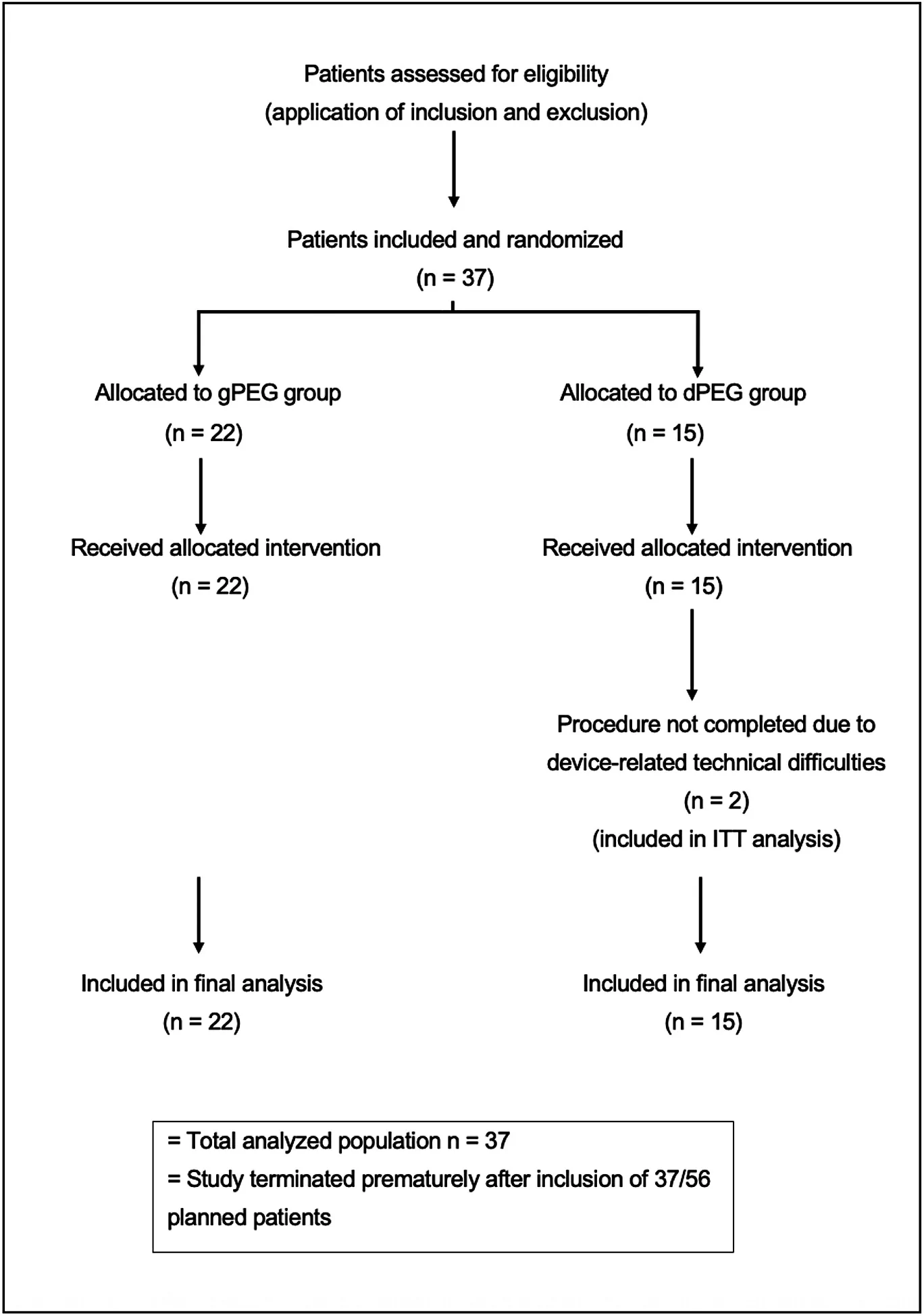
Flow diagram of patients undergoing randomized comparison of gastropexy-assisted PEG (gPEG) and introducer PEG without gastropexy (dPEG).

Fig. 1 summarizes patient allocation, intervention completion, and analysis from the point of eligibility confirmation and randomization. As no safety concerns were anticipated, no predefined termination criteria were established prior to the start of the study.

### Outcome Measures

The primary endpoint of the study was the PEG placement time, defined as the duration from local skin incision to successful intragastric positioning of the tube. This time was recorded in seconds using a stopwatch by an independent observer.

Secondary endpoints included the total procedure time, measured from endoscope insertion to completion of external fixation of the PEG system, also recorded in seconds. Further secondary outcomes encompassed the technical success rate, defined as successful PEG placement without the need for device exchange or procedural conversion.

Technical feasibility and system handling were systematically assessed by the performing endoscopist immediately after each procedure using a five-point grading scale: 1 – *Very easy placement*, trocar advanced without resistance; 2 – *Good placement*, trocar advanced with minimal resistance; 3 – *Satisfactory placement*, technically feasible with some effort; 4 – *Difficult placement*, substantial effort required to complete the procedure; 5 – *Placement not possible*, PEG could not be successfully inserted with the assigned device.

AEs were monitored and recorded for up to 30 days post procedure. Events were categorized by time of onset into acute (within 24 h), subacute (1–7 days), and delayed (8–30 days). Each AE was classified according to the Clavien–Dindo system. 5 Minor AEs were defined as Grade I (any deviation from the normal postoperative course not requiring intervention) or Grade II (requiring pharmacological treatment). Major AEs included Grade III (requiring surgical, endoscopic, or radiologic intervention) and Grade IV (life-threatening complications requiring intensive care management).

Additional secondary endpoints included PEG tube patency over the 30-day follow-up period, defined as continuous functional access without occlusion, and the rate of tube dislocation, defined as either internal or external displacement of the tube necessitating repositioning, replacement, or surgical intervention.

### PEG Procedure

All PEG procedures were performed in accordance with the current guidelines of the European Society of Gastrointestinal Endoscopy (ESGE). 6 Upper gastrointestinal endoscopy, including duodenoscopy, was conducted under conscious sedation using intravenous propofol 1% with supplemental oxygen administered via nasal cannula or tracheostomy tube. Continuous monitoring of heart rate, oxygen saturation, and blood pressure was carried out throughout the procedure in compliance with sedation safety guidelines. 7

Following insufflation of the stomach with carbon dioxide (CO₂) to achieve optimal gastric wall distension, the puncture site on the abdominal wall was identified using a combination of endoscopic transillumination and external palpation (finger indentation technique). The abdominal skin was then disinfected, and local anesthesia was administered by infiltrating 10 mL of a local anesthetic solution (Scandicain 1%) into all layers of the abdominal wall. All procedures were performed under aseptic conditions by experienced endoscopists with a minimum of seven years of independent endoscopic practice.

#### Introducer PEG with Gastropexy (gPEG)

For the gPEG, fixation of the stomach to the abdominal wall was achieved using two transabdominal sutures placed approximately 2 cm apart from the planned insertion site, employing a double-lumen gastropexy device (Freka Pexact II, Fresenius, Bad Homburg, Germany). Under endoscopic visualization, a skin incision of approximately 5 mm was made between the sutures, and a trocar with a peel-away sheath was advanced into the gastric lumen. After removal of the trocar, a balloon-retained feeding catheter (15 Charriere) was inserted through the sheath and secured via balloon inflation with 10 mL of sterile water. The sheath was subsequently peeled away, and the placement was finalized. Gastropexy sutures were removed between day 7 and 10 post-intervention.

#### Introducer PEG without Gastropexy (dPEG)

**Fig. 2:**
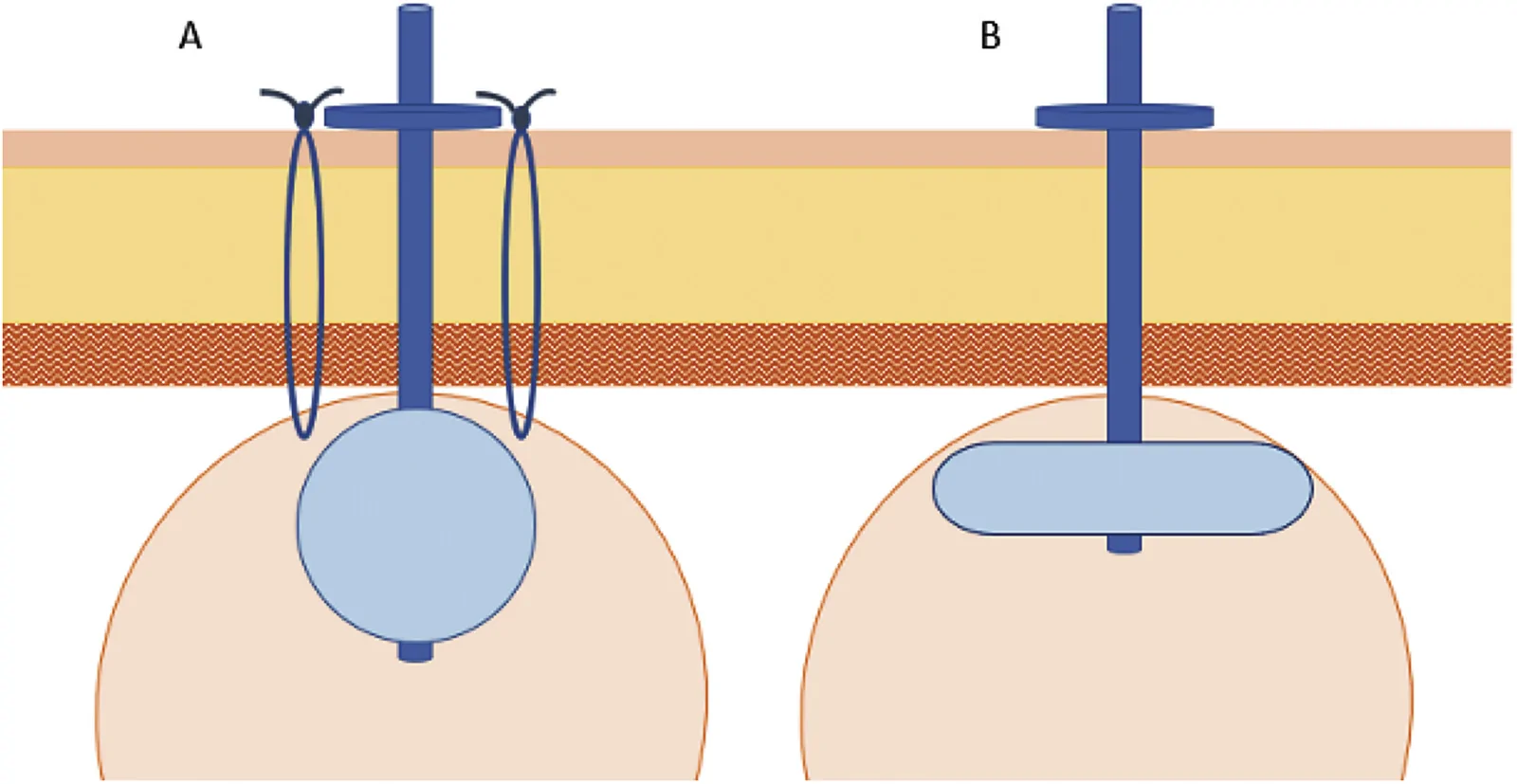
Schematic comparison of (A) introducer PEG with gastropexy sutures and (B) dPEG without sutures but flat, disc-shaped balloon.

All endoscopists received training in the new system from the company. In the dPEG group, following local anesthesia and skin incision, direct puncture of the stomach was performed using the dedicated introducer trocar included in the package (FLOCARE DirectPEG, Nutricia Milupa, Frankfurt, Germany). The feeding tube (15 Charriere) was then inserted directly through the trocar, and its tire-shaped internal retention balloon was inflated with 5 mL of sterile water. Proper tension and fixation were guided using the integrated color scale on the external bumper (tightened within the blue zone). The external bumper was subsequently loosened at 48 h post-placement (to the green zone) to reduce pressure on the abdominal wall and minimize tissue trauma ( Fig. 2 ).

### Follow-up

All patients were followed for a period of 30 days post-procedure to assess clinical outcomes and potential AEs. Clinical data, including AEs, mortality, tube dislocation, and tube patency, were initially collected from the hospital’s electronic medical records. In cases where no follow-up data were available within the institutional system, additional information was obtained through direct contact.

At 30 days post-intervention, either the patient or their primary care physician (general practitioner) was contacted by telephone. A structured follow-up interview was conducted to evaluate the presence of any AEs, including infections, dislocation or dysfunction of the feeding tube, and any relevant hospitalizations or medical interventions. Information regarding tube patency and the occurrence of death during the follow-up period was also recorded. This comprehensive follow-up ensured the capture of clinically relevant outcomes not documented during the initial hospital stay.

### Sample Size Calculation and Statistical Analysis

The sample size was calculated based on the study’s primary endpoint: time required for PEG placement. Based on clinical experience and existing literature, the mean total procedure time in the control group (gPEG) was estimated to be 450 s. The novel gastropexy-free dPEG system was hypothesized to reduce procedure time by 145 s. Assuming a standard deviation of 180 s in both groups, a two-sided significance level (α) of 0.05, and a statistical power (1–β) of 80%, the required sample size was calculated using the formula for comparison of two independent means. Based on this calculation, a minimum of 25 patients per group (50 total) was determined to be sufficient to detect the specified difference. To account for potential dropouts or incomplete follow-up data, the target sample size was increased by 10%, resulting in a final planned enrollment of 28 patients per group, for a total of 56 patients.

Sample size was calculated using G*Power calculation software, version 3.1.9.2 ( http://gpower.hhu.de ).

Descriptive data are presented as mean ± standard deviation unless otherwise specified. Comparative analyses were conducted using the Mann–Whitney *U* test for nonparametric comparisons of unpaired data, and Fisher’s exact test was applied for categoric variables where appropriate. A two-sided *p* -value of <0.05 was considered indicative of statistical significance. All statistical analyses were performed using GraphPad Prism, version 9.5.1 (GraphPad Software, La Jolla, CA, USA), under an institutional license for the University Medical Center Schleswig-Holstein.

## Results

Of the initially planned 56 patients, a total of 37 patients (66%) were enrolled due to premature termination of the study. Instead of the intended allocation of 28 patients per group, 22 patients (59%) were assigned to the gastropexy-assisted PEG group and 15 patients (41%) to the introducer PEG without gastropexy group. In two patients (13%) in the dPEG group, PEG placement could not be completed due to technical device-related difficulties. In accordance with the intention-to-treat principle, these patients were included in the final analysis.

Baseline patient characteristics were comparable between the groups with respect to age, sex, ASA classification, underlying disease, and indication for PEG placement. A statistically significant difference was observed only in body mass index (BMI), which was lower in the dPEG group compared with the gPEG group (20.56 vs. 25.48; *p* = 0.01). Detailed patient characteristics and indications are summarized in Table 1 .

**Table 1:** Patient characteristics and indications for PEG

	Total	gPEG group	dPEG group	*p* -Value
Number of patients [ *N* ] (%)	37	22	15	
Female [ *N* ] (%)	8 (21.6)	5 (22.7)	3 (20)	> 0.99
Age [years] (mean ± SD)	68.11 ± 9.4	69.18 ± 9.67	66.53 ± 9.05	0.41
BMI (mean ± SD)	23.49 ± 5.7	25.48 ± 6.1	20.56 ± 3.6	0.01
ASA-classification [ *N* ] (%)				
ASA 1	0	0	0	
ASA 2	17 (45.9)	11 (50)	6 (40)	0.74
ASA 3	20 (54.1)	11 (50)	9 (60)	0.74
ASA 4	0	0	0	
Underlying disease				
Malignant	34	20	14	>0.99
Neurologic	3	2	1	>0.99
Leading indication				
Before radiation	11	7	4	>0.99
Dysphagia	26	15	11	>0.99

**Fig. 3:**
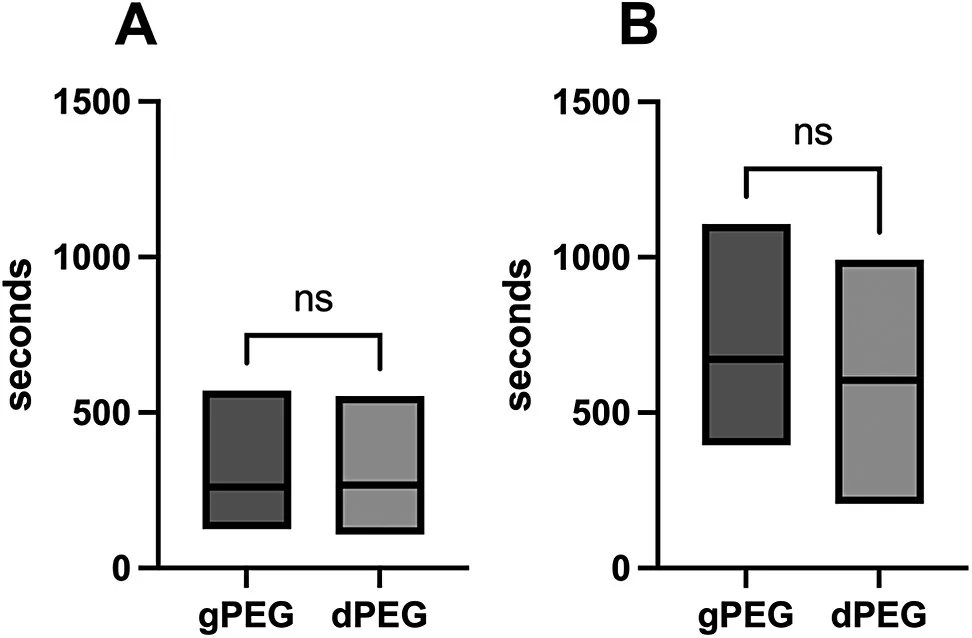
Comparison of (A) PEG placement time and (B) total procedure time between gPEG and dPEG.

The mean procedure for PEG placement time was 260 ± 109 s in the gPEG group and 265 ± 145 s in the dPEG group, with no statistically significant difference between the groups ( *p* = 0.91), thereby not meeting the primary endpoint. Similarly, the total procedure time did not differ significantly (671 ± 200 s vs. 604 ± 232 s; *p* = 0.38; Fig. 3 ).

**Fig. 4:**
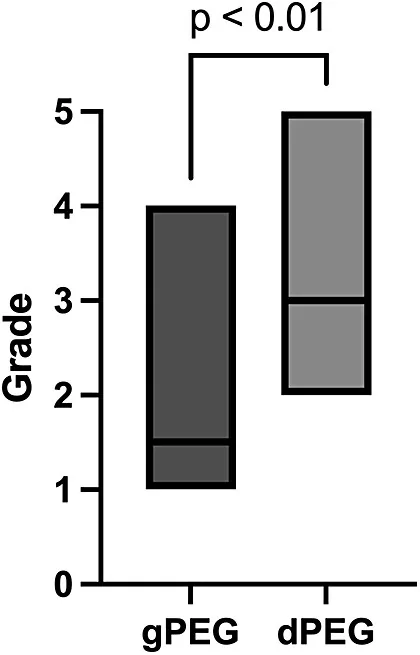
Grading of technical feasibility.

**Fig. 5:**
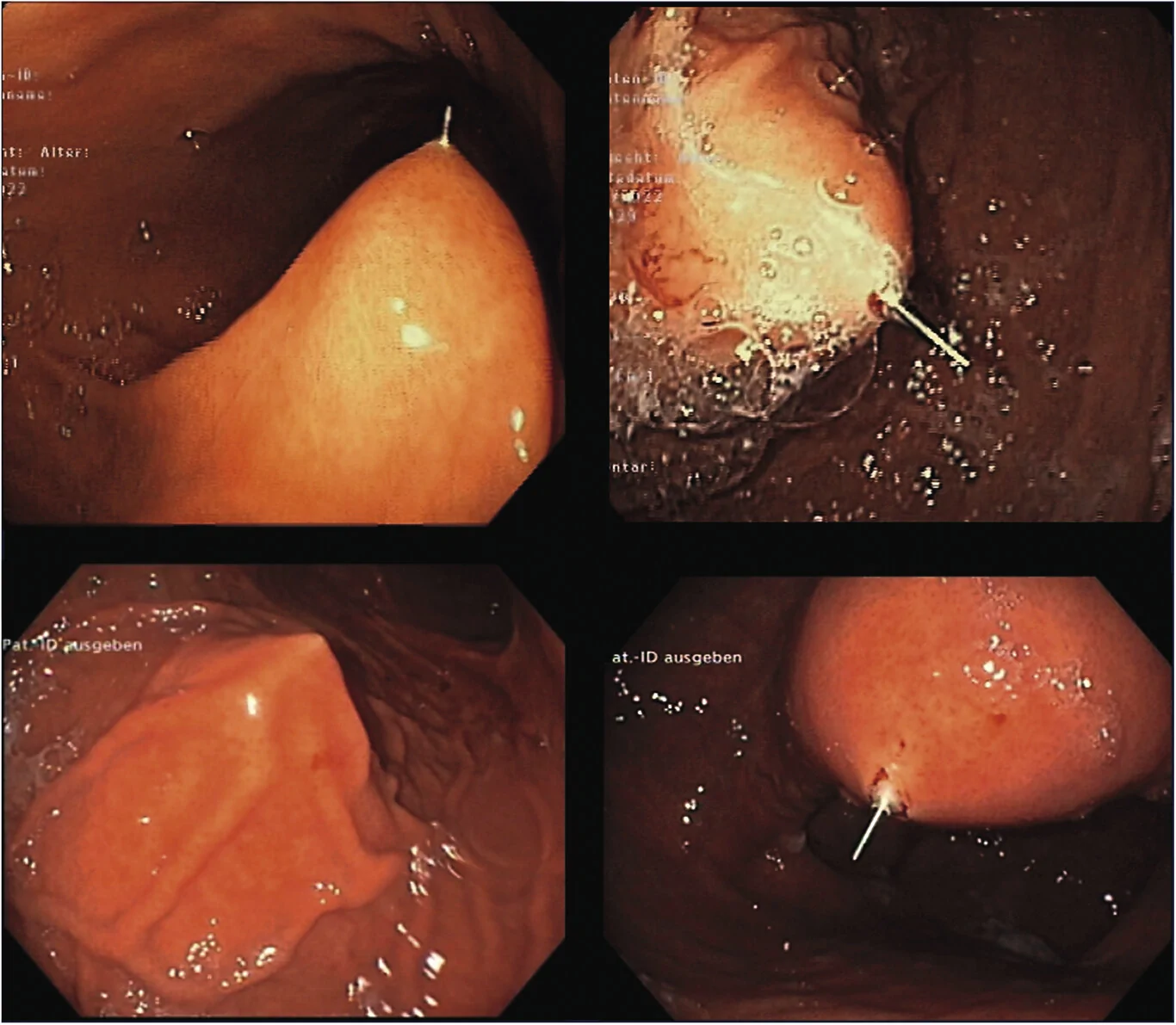
Examples of “tenting phenomenon” during dPEG placement.

Technical feasibility, as rated by the endoscopist on a 5-point grading scale (1 = best performance, 5 = worst performance), was significantly superior in the gPEG group compared with the dPEG group (median score 1.5 [25–75% IQR: 1-2] vs. 3.0 25–75% IQR 2–4]; *p* < 0.01; Fig. 4 ). A major contributor to the inferior technical performance of dPEG was difficulty during trocar insertion through the gastric wall. In several cases, the stomach wall was displaced anteriorly without adequate penetration, a phenomenon referred to as the “tenting phenomenon” ( Fig. 5 ). Successful advancement of the trocar with peel-away sheath frequently required substantial force and repeated manipulation. Additionally, adjustment of the puncture angle under endoscopic visualization was often necessary to avoid contact of the trocar with the posterior gastric wall. In two cases, the procedure was aborted due to failure of trocar advancement and concern for potential collateral injury.

**Fig. 6:**
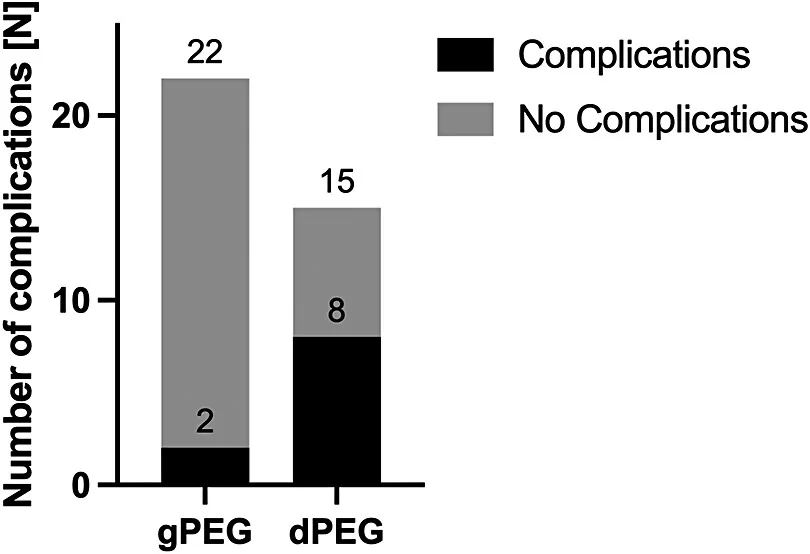
Overall complications.

Within the 30-day follow-up period, acute complications occurred in 2 patients (9%) in the gPEG group and in 8 patients (53%) in the dPEG group ( *p* = 0.0064; Fig. 6 ), corresponding to a risk difference of 0.44 (95% CI 0.13–0.76) and a relative risk of 5.87 (95% CI 1.42–24.2). All complications were classified as minor. Localized pain requiring analgesic treatment (Clavien–Dindo grade I) was observed in both patients in the gPEG group and in five patients in the dPEG group. One patient in the dPEG group developed localized peritonitis associated with elevated inflammatory markers, which was successfully treated with antibiotics (Clavien–Dindo grade II). No major complications (Clavien–Dindo grade ≥ III) were observed.

Tube dislocation occurred in 5 patients (33%) in the dPEG group compared with 2 patients (9%) in the gPEG group, representing a numerical increase without reaching statistical significance ( *p* = 0.093). The corresponding risk difference was 0.24 (95% CI −0.003 to 0.49). During the 30-day follow-up period, two patients in the gPEG group died due to progression of their underlying disease; these events were deemed unrelated to the PEG procedure. No deaths occurred in the dPEG group.

Given the repeated technical difficulties associated with trocar insertion, primarily attributable to the “tenting phenomenon,” and notably more frequent than expected clinical reports of post-procedural pain from patients in the dPEG group, a systematic evaluation of all patients enrolled up to that time was undertaken. This analysis was initiated in response to these emerging clinical observations rather than as a predefined interim analysis. The results of this evaluation supported the initial clinical impression, demonstrating a trend toward differences in procedural outcomes between the two groups. Based on these findings and the ongoing assessment of the risk–benefit profile, the study was prematurely discontinued after inclusion of 37 patients. The results presented in this manuscript therefore represent the final analysis of all available data collected during the study period. The manufacturer was subsequently contacted to discuss potential technical modifications of the device.

## Discussion

The aim of this study was to compare the novel gastropexy-free introducer dPEG system (FLOCARE DirectPEG, Nutricia Milupa, Frankfurt, Germany) with the established gastropexy-assisted gPEG system (Freka Pexact II, Fresenius, Bad Homburg, Germany), representing, to our knowledge, the first direct comparison of these two techniques. By eliminating the need for gastropexy sutures, the dPEG system was theoretically expected to simplify the procedure, reduce placement time, and potentially decrease procedure-related complications such as pain. 8 However, our findings did not confirm these anticipated advantages. While PEG placement time and overall procedure duration were comparable between groups, dPEG insertion was associated with reduced technical feasibility and a trend toward a higher rate of procedure-related complications. Following increasing clinical concerns during the study period, an interim analysis was performed, and the study was terminated prematurely after inclusion of 37 patients rather than the initially planned 56 patients.

Previous studies have reported total procedure times for gPEG placement ranging from 10 to 23 min. 9 10 11 In our study, both PEG placement time and total procedure time were comparable between groups, with mean procedure durations of approximately 11 min for gPEG and 10 min for dPEG, respectively, representing the lower range of previously reported values. Although the dPEG system was designed to reduce procedural duration by avoiding the initial gastropexy steps, this theoretical benefit was not observed. The primary endpoint of a reduction in PEG placement time was therefore not achieved.

An imbalance in baseline characteristics was observed regarding BMI, with higher BMI values in the gPEG group. BMI may influence PEG placement by affecting abdominal wall thickness, fixation conditions, and technical complexity. Therefore, the higher BMI in the gPEG group could theoretically have represented a disadvantage for this technique. Nevertheless, despite this potential bias, PEG placement times were comparable between groups, and the dPEG group demonstrated a higher rate of procedure-related complications. Thus, the BMI difference is unlikely to fully account for the observed differences in outcome, although it remains a relevant confounding factor that should be considered when interpreting these findings.

The prolonged insertion process observed in the dPEG group was primarily related to the so-called “tenting phenomenon,” which represented a major technical challenge during trocar advancement. Instead of penetrating the gastric wall, the applied force frequently resulted in anterior displacement of the stomach, preventing controlled advancement of the trocar and peel-away sheath. Consequently, repeated adjustments of the puncture angle under endoscopic guidance were required to achieve successful access while minimizing the risk of injury to the posterior gastric wall.

Effective apposition of the gastric wall to the abdominal wall is considered an important prerequisite for safe and controlled PEG placement. A study from Brazil evaluated a novel introducer PEG approach combined with T-anchor fixation and reported shorter insertion times compared with the conventional pull technique (6.4 vs. 12.6 min). 12 However, these findings originate from single-center experiences and should be interpreted cautiously. Similarly, retrospective comparative studies have suggested that gastropexy-assisted PEG techniques may be associated with lower rates of major complications compared with conventional pull-through PEG placement. 1 These observations support the hypothesis that prior gastric fixation may improve procedural control by stabilizing the gastric wall and reducing the risk of malposition or intraperitoneal complications. However, the current evidence supporting these potential advantages is predominantly based on retrospective studies, with only limited prospective randomized data available.

Reported complication rates for introducer PEG vary widely from 0% to 48%. 2 3 8 10 11 13 Major complications are uncommon and are most frequently related to respiratory failure. 3 Post-procedural pain represents the most commonly reported AE, with reported incidences ranging from 3.6% to 60%, depending in part on PEG tube diameter. 13 Although gastropexy sutures have been discussed as a potential contributor to local irritation and pain, comparative studies between pull-through and introducer PEG techniques have demonstrated comparable rates of post-procedural pain, suggesting that gastropexy itself does not necessarily increase pain. 2 3 In our study, both techniques demonstrated an overall favorable safety profile without major complications. However, contrary to the initial hypothesis that avoiding gastropexy might reduce local pain, significantly higher post-procedural pain was observed in the dPEG group. This finding suggests that factors beyond the presence of gastropexy sutures may influence pain development. In particular, the technically challenging insertion process associated with the “tenting phenomenon” may have contributed to increased tissue trauma and patient discomfort. The required application of force and repeated manipulation of the gastric wall during advancement of the peel-away sheath may have impaired controlled device placement. Furthermore, insufficient counterpressure within the stomach during CO₂ insufflation may have resulted in displacement between the gastric and abdominal walls, potentially facilitating minor leakage of gas or gastric contents into the peritoneal cavity and contributing to localized irritation. From a procedural perspective, the limited control during trocar advancement and uncertainty regarding device positioning may have contributed to the reduced technical feasibility observed with the dPEG system.

In our cohort, the dPEG group showed a numerically higher dislocation rate compared with the gPEG group, without reaching statistical significance. The observed dislocation rate was also higher than previously reported rates for introducer PEG systems; however, this finding should be interpreted cautiously given the limited sample size and differences between study populations. 3

**Fig. 7:**
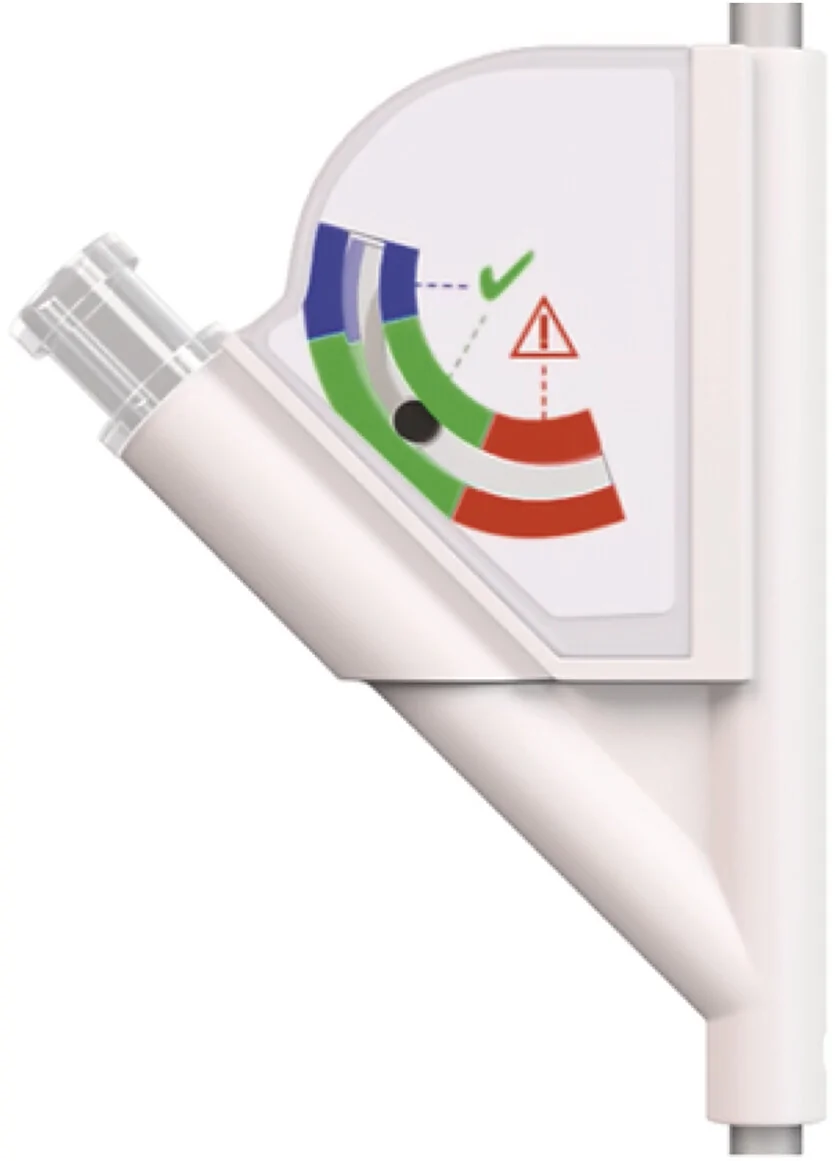
Balloon indicator with the different areas: red (pressure too low), green (optimum pressure), and blue (pressure too high/optimum pressure after installation).

This finding was most likely related to handling and management challenges during the early implementation phase rather than an inherent device failure. Although patients and nursing staff received prior training, practical difficulties emerged during routine care. The dPEG system includes a visual indicator reflecting intragastric balloon pressure, which is designed to remain within a defined optimal range (green zone, Fig. 7 ). During inpatient follow-up, a decrease in the indicator level was frequently observed. Importantly, this did not reflect balloon leakage or loss of filling volume but was primarily caused by loosening of the external fixation plate, resulting in reduced apposition of the balloon against the gastric wall. In several cases, the indicator change was misinterpreted as insufficient balloon filling, leading to unnecessary balloon refilling. This subsequently contributed to balloon rupture and tube dislocation. After recognition of this issue, the manufacturer modified the fixation plate to improve tube stabilization, and additional targeted training was implemented to optimize handling of the system.

This study has several limitations that should be considered when interpreting the results.

First, premature termination following interim analysis led to a substantially reduced sample size compared with the original calculation, thereby limiting the statistical power of the study and the generalizability of the findings. In particular, the imbalance in group sizes and the overall small cohort may have affected the robustness of subgroup comparisons and secondary endpoints.

Second, no adjustment for multiple testing of secondary endpoints was performed. Therefore, the results of secondary analyses should be interpreted cautiously and considered hypothesis-generating rather than confirmatory.

Third, this was a single-center study, and the observed technical challenges and complication rates may, to some extent, reflect local procedural experience or the early adoption phase of a novel device. Although all operators and nursing staff received structured training, a learning curve effect cannot be excluded and may have contributed to both technical difficulties and handling-related AEs. Furthermore, technical feasibility was evaluated using a five-point rating scale specifically developed for this trial, which has not yet been externally validated. This assessment was inherently subjective and may have been influenced by the lack of operator blinding and by individual experience with the respective techniques. Although this approach was chosen to capture practical aspects of device handling and procedural performance in a real-world setting, the results should be interpreted with caution.

Fourth, the investigational device underwent evaluation during an early phase of clinical implementation and was subsequently withdrawn from the market for further technical refinement. As a result, the findings primarily reflect the performance of an early device iteration and may not be fully transferable to future, optimized versions of the system.

Despite these limitations, the study provides important prospective randomized data highlighting relevant safety concerns and technical challenges, which were instrumental in informing the manufacturer’s decision to suspend distribution and further improve the device.

## Conclusion

In summary, established gastropexy-assisted techniques remain the preferred approach for PEG placement. The anticipated procedural advantages of omitting gastropexy were not observed, while higher rates of technical difficulties and AEs were evident in the dPEG group.
